# Supplementary material for: 3D printing of self-healing longevous multi-sensory e-skin
Source: Commun Mater. 2025 Jun 13;6(1):121. doi: 10.1038/s43246-025-00839-7 (PMC12165852; doi:10.1038/s43246-025-00839-7)
Supplement: Supplementary file 2 — Supporting Information [file 43246_2025_839_MOESM2_ESM.pdf]

Supplementary Information

3D Printing of Self-healing Longevous Multi-sensory E-Skin

Antonia Georgopoulou[1,2], *Sudong Lee*[3], *Benhui Dai*[3], *Francesca Bono*[1], *Josie Hughes*[3],

Esther Amstad[1,2*]

1) Soft Materials Laboratory, Institute of Materials (SMaL), École Polytechnique Fédérale de Lausanne, 1015 Lausanne, Switzerland.

2) Swiss National Center for Competence in Research (NCCR) Bio-inspired Materials, University of Fribourg, Chemin des Verdiers 4, 1700 Fribourg, Switzerland.

3) CREATE Lab, Institute of Mechanical Engineering, École Polytechnique Fédérale de Lausanne, 1015 Lausanne, Switzerland.

E-mail: esther.amstad@epfl.ch

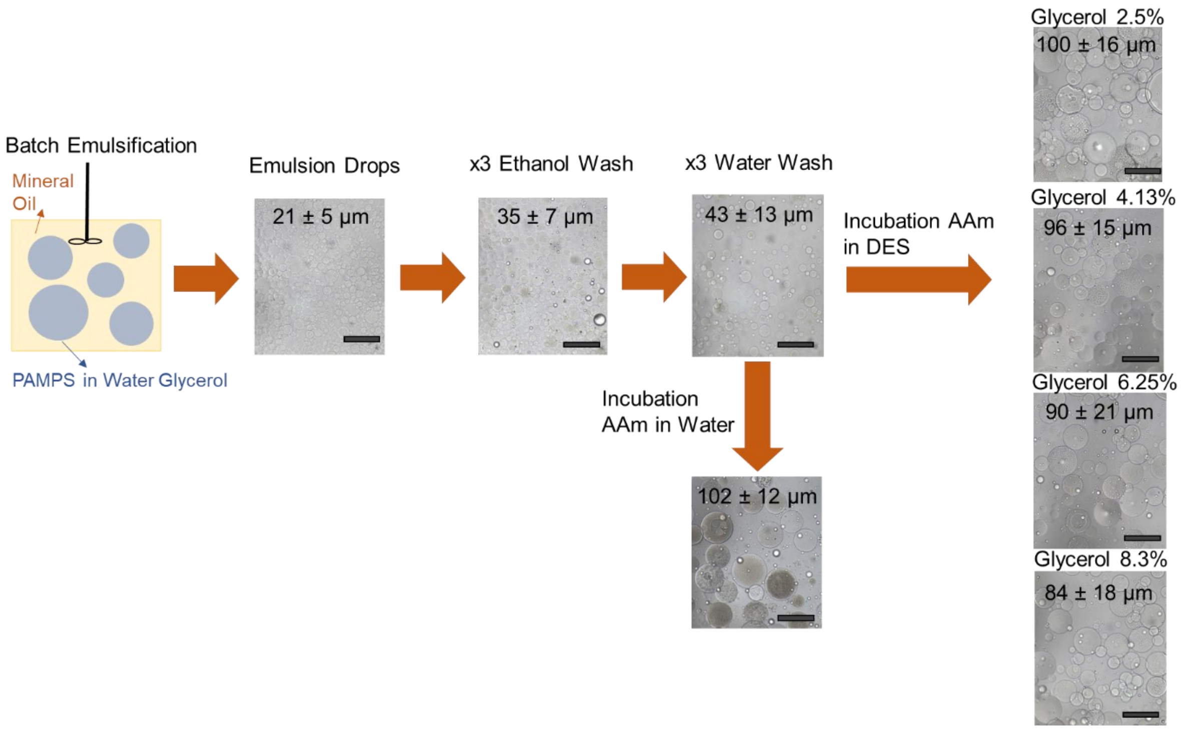

Supplementary Figure 1. Optical micrographs of the drops and resulting microgels during the different preparation stages starting from the batch emulsification and ending with the final incubation in water or a DES solution. The average diameters of the drops and microparticles are indicated on top of the images. Scale bars (100 µm).

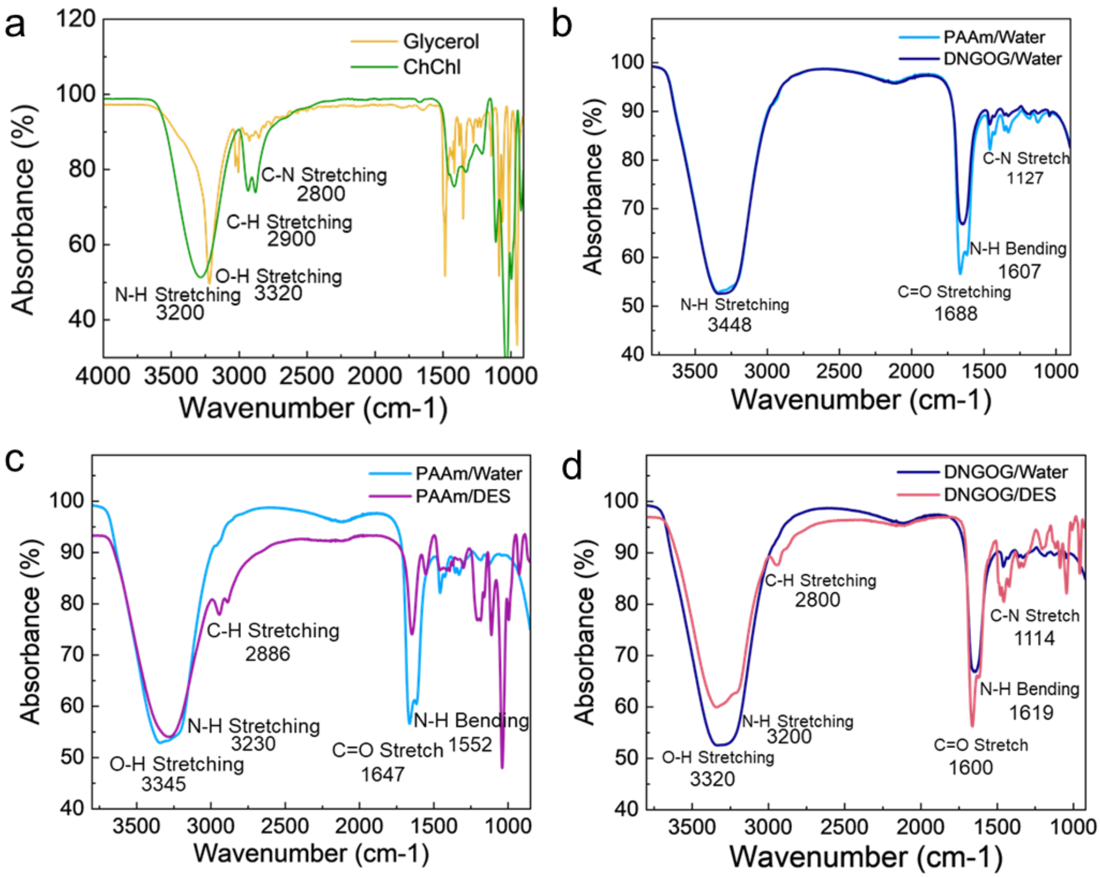

Supplementary Figure 2. FTIR spectra of a) glycerol (yellow) and choline chloride (green) b) PAAm (blue) and DNGOG (navy) and c) PAAm (blue) swollen with water and PAAm functionalized with DES (purple) d) DNGOG swollen with water (navy) and DNGOG functionalized with DES (red).

The PAAm displays characteristic peaks at 3370 cm^{-1} and 3166 cm^{-1} that we assign to the asymmetric and symmetric vibrations of the amine (N-H) bond, 1662 cm^{-1} assigned to the bending vibration of the carbonyl group (C=O), 1610 cm^{-1} the bending vibration of amine (N-H), 2810 cm^{-1} and 1467 cm^{-1} are assigned to the stretching and bending vibrations of the methyl (C-H) groups, respectively. PAMPS and AAm possess amine and carboxyl peaks. In addition to these vibrations, PAMPS displays characteristic peaks at 1235 cm^{-1} and 1042 cm^{-1} corresponding to the asymmetric and symmetric S-O stretching of the sulfonate group. The DNGOG consisting

of PAMPS, PAAm and DES revealed similar spectra as the individual components, showing characteristic peaks of amide, carboxyl, sulfonate groups and we do not observe any shifts of these peaks. Upon addition of the DES to single-network PAAm, we observe a prominent peak at 2935 cm^{-1} that we assign to the C-N stretch vibration, characteristic of the ammonium group of the choline chloride, as shown in Figure S3a. In addition, the characteristic peak of the sulfonate appears at 1042 cm^{-1} for the DNGOG. This peak does not significantly shift upon addition of the DES, as shown in Figure S3b.

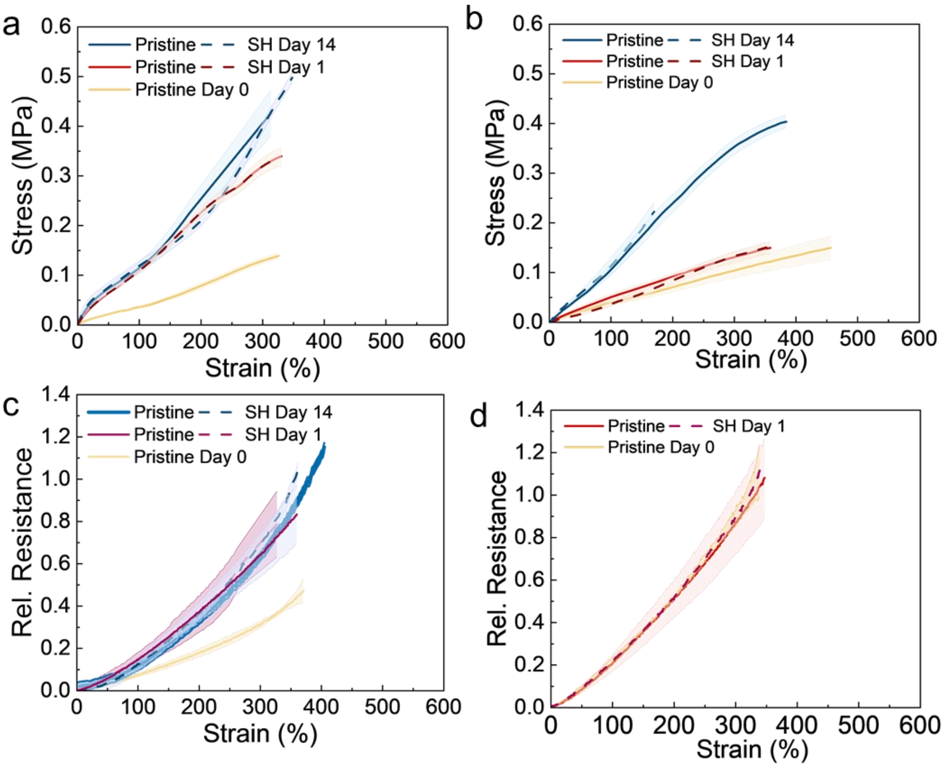

Supplementary Figure 3. a) Tensile tests of DNGOGs containing (a) 2.5% and (b) 8.3 mol% glycerol at day 0 (yellow), day 1 (red), day 14 (blue) after production. c) Relative resistance-strain response of DNGOGs containing c) 2.5% and d) 8.3% glycerol after day 0 (yellow), day 1 (red), day 14 (blue). The DNGOGs with 8.3% glycerol did not exhibit self-healing behaviour after 14 days. The continuous lines symbolise the pristine samples and the dotted lines samples after having been damaged and self-healed by putting the two parts in contact for 10 s at 25°C, RH=40%.

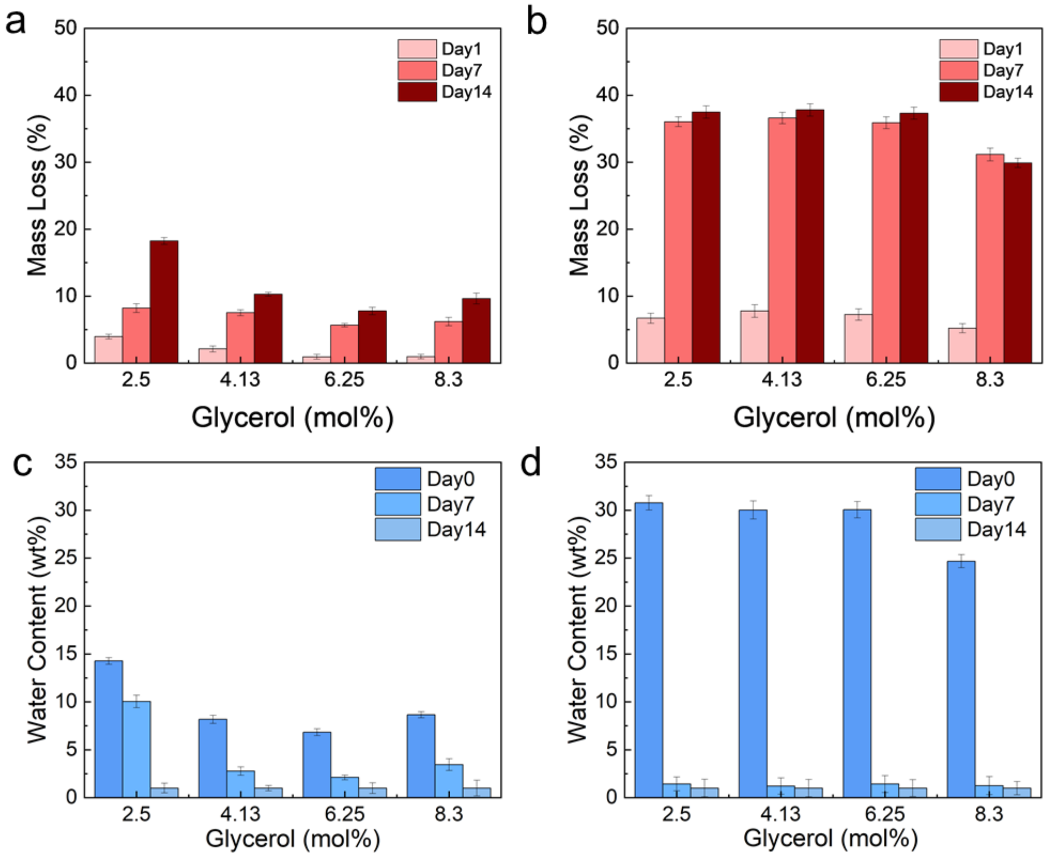

Supplementary Figure 4. Mass loss for a) PAMPS /PAAm double network bulk hydrogels and b) DNGOGs as a function of the storage time and amount of glycerol contained in the DES. Water content for c) PAMPS /PAAm double network bulk hydrogels and d) DNGOGs as a function of the storage time and amount of glycerol contained in the DES.

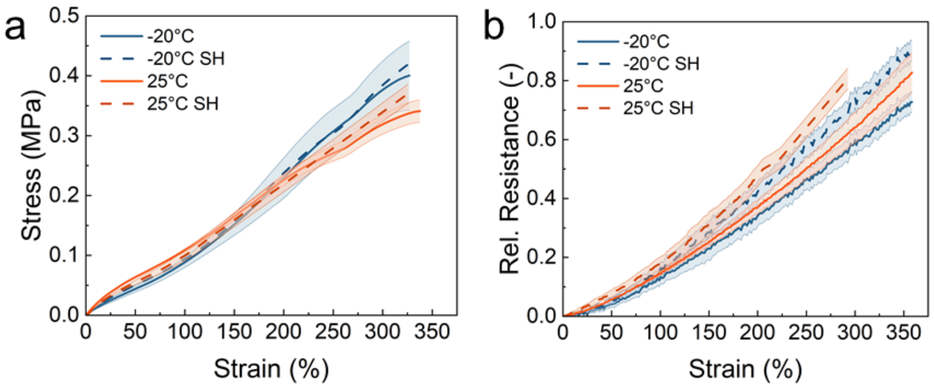

Supplementary Figure 5. a) Stress-strain response and b) relative resistance-strain response of the DNGOG with 2.5 mol% Glycerol at -20°C (blue) and 25°C (red). The continuous line symbolizes the response of the pristine sample and the dotted line that of broken samples after they have been put in contact for 10 s at RH=40%.

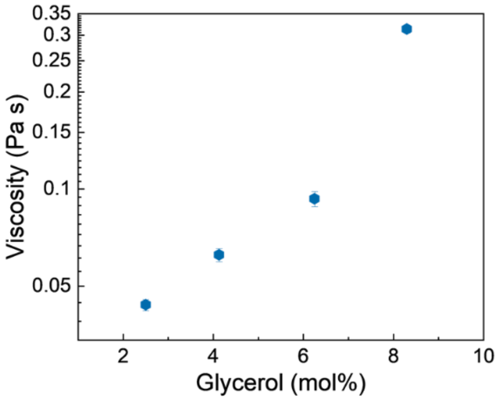

Supplementary Figure 6. Viscosity of DES as a function of the glycerol concentration.

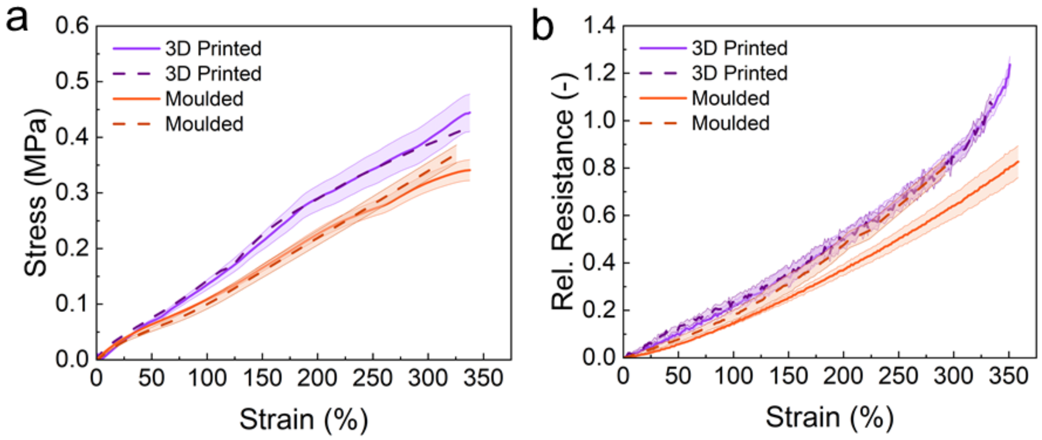

Supplementary Figure 7. a) Stress-strain response and b) resistance-strain response of the DNGOG with 2.5 mol% Glycerol after cutting the samples in half and self-healing after they have been put in contact for 10 s at RH=40% one time (red) and ten times (blue).

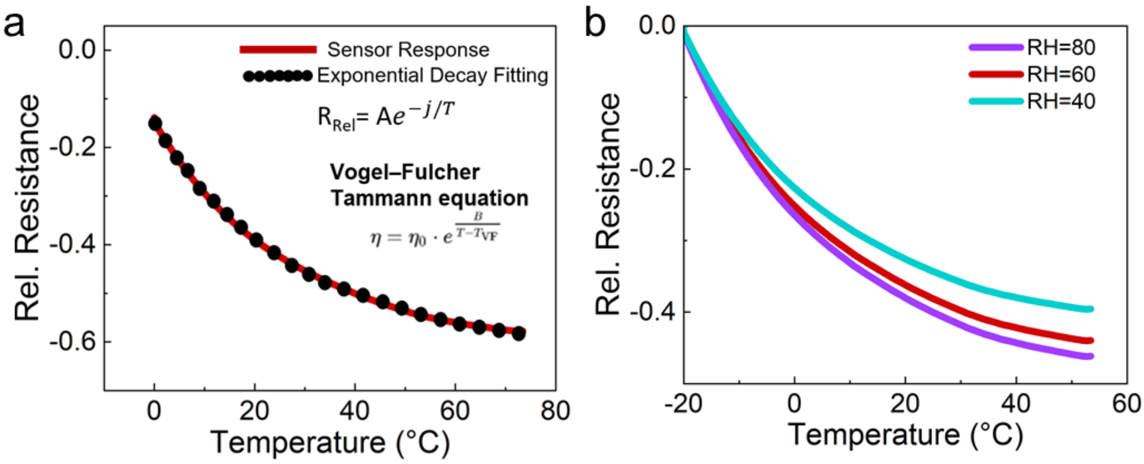

Supplementary Figure 8. a) Exponential decay fitting of the temperature-dependent relative resistance of DNGOGs containing 2.5% glycerol, measured at RH=80%. b) Relative resistance response with temperature at relative humidity 40% (blue), 60% (red) and 80% (violet).

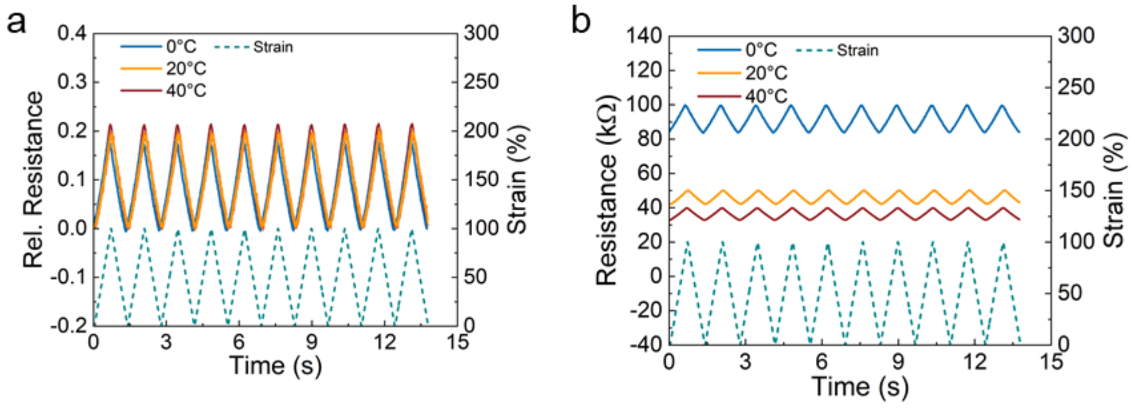

Supplementary Figure 9. Influence of temperature cycles on the resistance. a) The temperature was cycled by 10K starting from 0°C (green), 20°C (orange) and 40°C (red). All samples contained 2.5 mol% glycerol. b) The relative resistance-strain response of DNGOGs containing 2.5 mol% glycerol subjected to dynamic alterations in strain between 0 and 100% measured at 0°C (blue), 20°C (orange) and 40°C (red).

Supplementary Table 1. Formulation of DNGOGs with varying DES concentrations

	PAMPS Microgels	Acrylamide	Glycerol	Choline Chloride	MBA	PI*
	mol (%)	mol (%)	mol (%)	mol (%)	mol (%)	mol (%)
DES10	0.14	7.5	5	5	0.02	0.9
DES12.5	0.14	10	6.25	6.25	0.03	0.9
DES15	0.14	12.5	7.5	7.5	0.04	0.9

*Photoinitiator: 2-hydroxy-2-methylpropriophenone

Supplementary Table 2. Formulation of DNGOGs with varying molar ration between glycerol and choline chloride

	PAMPS Microgels	Acrylamide	Glycerol	Choline Chloride	MBA	PI*
	mol (%)	mol (%)	mol (%)	mol (%)	mol (%)	mol (%)
Gly-2.5	0.14	10	2.5	10	0.03	0.9
Gly-4.13	0.14	10	4.13	8.3	0.03	0.9
Gly-6.25	0.14	10	6.25	6.25	0.03	0.9
Gly-8.3	0.14	10	8.3	4.2	0.03	0.9

*Photoinitiator: 2-hydroxy-2-methylpropriophenone

Supplementary Table 3. Tensile properties before damage and after self-healing by putting the two parts in contact for 10 s at 25°C, RH=40% for DNGOGs with glycerol concentration 2.5 mol% (Gly-2.5) and 8.3 mol% (Gly-8.3). These data correspond to the plots depicted in Figure S2.

Sample	Young's Modulus Before Damage (kPa)	Young's Modulus After Healing (kPa)	Ultimate Strength Before Damage (kPa)	Ultimate Strength After Healing (kPa)	Strain at Break Before Damage (kPa)	Strain at Break After Healing (kPa)
Gly-2.5 Day 1	138	135	339	340	330	332
Gly-2.5 Day 14	152	149	436	487	321	340
Gly-8.3 Day 1	53	40	352	352	358	352
Gly-8.3 Day 14	109	112	403	221	384	170

Supplementary Table 4. Recovery of the Young's Modulus after self-healing by putting the two parts in contact for 10 s at 25°C, RH=40% for DNGOGs with glycerol content 2.5 mol% (Gly-2.5), 4.13 mol% (Gly-4.13), 6.25 mol% (Gly-6.25) and 8.3 mol% (Gly-8.3).

Sample	Recover of Young's Modulus (%)		
	Day 0	Day 1	Day 14
Gly-2.5	1.2 ± 0.02	98 ± 2	96 ± 3
Gly-4.13	0.8 ± 0.02	86 ± 3	85 ± 2
Gly-6.25	0.5 ± 0.04	86 ± 1	86 ± 3
Gly-8.3	0.5 ± 0.02	75 ± 2	75 ± 1

Supplementary Table 5. Recovery of the electrical resistivity after self-healing by putting the two parts in contact for 10 s at 25°C, RH=40% for DNGOGs with glycerol content 2.5 mol% (Gly-2.5), 4.13 mol% (Gly-4.13), 6.25 mol% (Gly-6.25) and 8.3 mol% (Gly-8.3).

Sample	Recover of Electrical Resistivity (%)		
	Day 0	Day 1	Day 14
Gly-2.5	2 ± 0.01	124 ± 5	115 ± 5
Gly-4.13	4 ± 0.02	120 ± 8	105 ± 5
Gly-6.25	5 ± 0.01	105 ± 5	-
Gly-8.3	4 ± 0.01	98 ± 3	-
