# Supplementary material for: 3D printing of self-healing longevous multi-sensory e-skin
Source: Commun Mater. 2025 Jun 13;6(1):121. doi: 10.1038/s43246-025-00839-7 (PMC12165852; doi:10.1038/s43246-025-00839-7)
Supplement: Supplementary file 3 — Description of additional supplementary files [file 43246_2025_839_MOESM3_ESM.pdf]

Description of Additional Supplementary Files

File name: Supplementary Movie 1.

Description: Testing the multimodal response of 3D printed e-skin wearables by recording the electrical resistance during pressing a keyboard button, immersing the e-skin in water, approaching the surface of ice and a mug filled with hot tea with the e-skin.
